# Supplementary material for: Cj0683 Is a Competence Protein Essential for Efficient Initialization of DNA Uptake in Campylobacter jejuni
Source: Biomolecules. 2023 Mar 11;13(3):514. doi: 10.3390/biom13030514 (PMC10046745; doi:10.3390/biom13030514)
Supplement: Supplementary file 1 [file biomolecules-13-00514-s001.zip › biomolecules-2135083-supplementary.pdf]

Supplementary Material

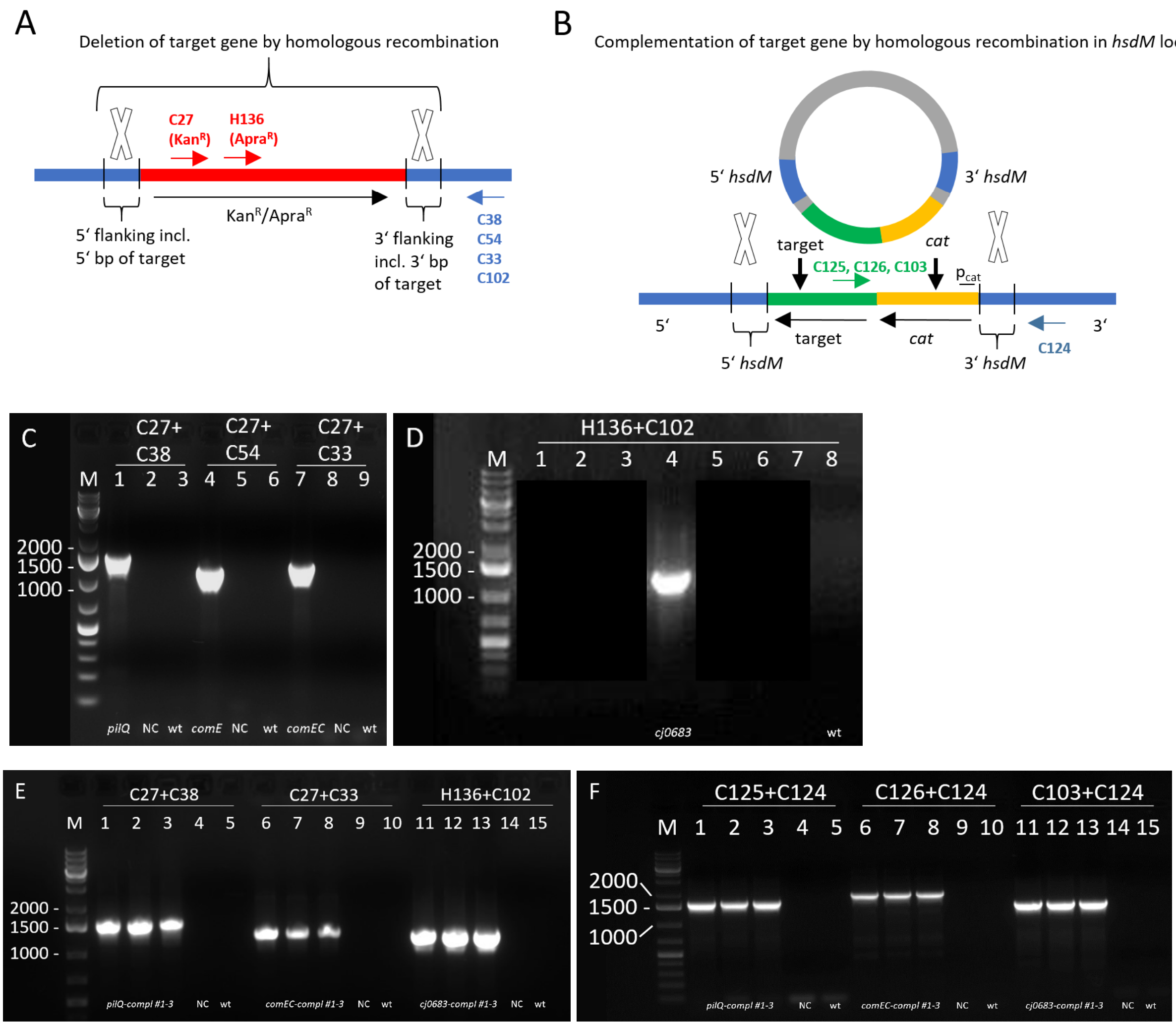

Figure S1. PCR results of successful allelic exchange of mutant strains used in the study. **A,** Cloning strategy for construction of deletion mutants and **B**, their complemented strains. Kan^R, kanamycin resistance cassette; $Apra^R$, apramycin resistance cassette; *cat*, chloramphenicol resistance cassette including the promoter (p_{cat}). Flanking regions include 6 to 123 bp of the respective target gene. PCR fragments were separated on a 1 % agarose gel (**C-E**). Lanes are numbered above and annotated below with the respective mutant name, wt (wild type), NC (negative control) or M (1 kb DNA ladder). Other non-relevant lanes are covered by black boxes. **C**, PCR results for verification of successful deletion of *pilQ* (C27+C38), *comE* (C27+C54), and *comEC* (C27+C33). **D**, PCR results for verification of successful deletion of *cj0683* (H136+C102). **E**, PCR results for verification of successful deletion of the target gene at its native location in the complemented strains *pilQ-compl* (C27+C38), *comEC-compl* (C27+C33), and *cj0683-compl* (H136+102); three clones each are depicted (#). **F**, PCR results for verification of successful integration of the target genes in the complemented strains *pilQ-compl* (C125+C124), *comEC-compl* (C126+C124), and *cj0683-compl* (C103+C124) in *hsdM*; three clones each are depicted (#).

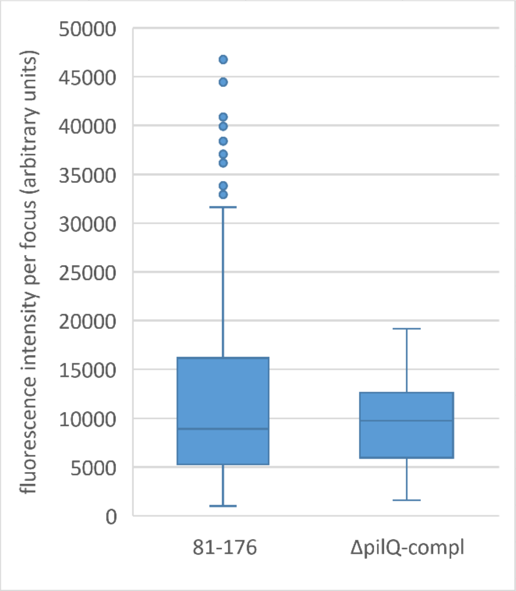

Figure S2. Median DNA amounts imported by Δ*pilQ*-compl (n=27) were comparable to the wild type (n=568). The boxplot length corresponds to the interquartile range (IQR; 50 %) of data, the horizontal bar indicates the median value; whiskers represent 1.5 × IQR or the maximum/minimum value of the dataset; dots, outliers.

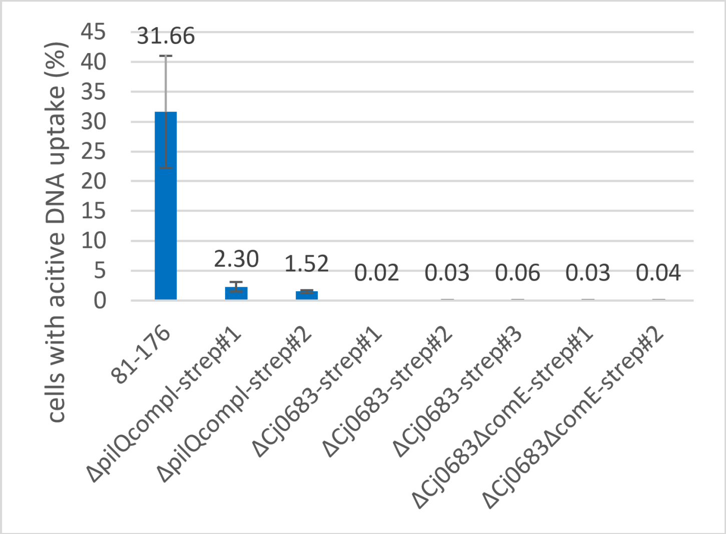

Figure S3. Successfully transformed mutant strains Δ*pilQ*-compl, Δ*cj0683*, and Δ*cj0683*Δ*comE* (name extension "–strep") displayed similar fractions of competent cells as their respective parental strains. Fraction of cells with functional DNA uptake after incubation with Cy3-labelled *C. jejuni* DNA is depicted. #, clone number. Results of three independent experiments are shown.

Table S1. Strains used in this study.

Strain	Reference	Species	Description
81-176	[31]	Cj	wild type strain
NCTC 11168	[35]	Cj	wild type strain
81-176Δ*pilQ*	This study	Cj	Mutant strain lacking of the outer membrane pore PilQ (=CtsD=Cj1474c), *pilQ*::Kan^R
81-176Δ*comE*	This study	Cj	Mutant strain lacking ComE (=Cj0011c), *comE*::Kan^R
81-176Δ*comEC*	This study	Cj	Mutant strain lacking the inner membrane channel ComEC (=Cj1211), *comEC*::Kan^R
81-176Δ*cj0683*	This study	Cj	Mutant strain lacking the periplasmic protein Cj0683, Δ*cj0683*::Apra^R
81-176Δ*pilQ*-compl	This study	Cj	Complemented strain of *pilQ*
81-176Δ*comEC*-compl	This study	Cj	Complemented strain of *comEC*
81-176Δ*cj0683*-compl	This study	Cj	Complemented strain of *cj0683*
DH5α-pSB3021	[37]/this study	Ec	Suicide vector for integration of target gene in *hsdM* for complementation
J99	[32]	Hp	wild type strain

Cj *Campylobacter jejuni*; Ec *Escherichia coli*; Hp *Helicobacter pylori*

Table S2. Oligonucleotides used in this study.

Name	Sequence (5′-3′)
C27	GGACATGATGCTATGGCTG
C29	TTGGATCCGGTTGGAATTTTAGCTGCAG
C30	CACCCGGGTACCGAGGAGAAAAATAAATAATGAAATTCTTTAAAAG
C31	TTTTAGTACCTGGAGGGAATAATGGAACTAATATCCATATCCCATTTTG
C32	TTTGTCGACCCAACAAACTTTAGATGATTATAAAATAG
C33	GCAACAAGATGTTGTTGCTAC
C34	TTGGATCCGCAATAAAAAAGACGCTCAAC
C35	CACCCGGGTACCGAGCGTTAATACTGATATCAAAAAGACG
C36	TTTTAGTACCTGGAGGGAATAATGGATAGCGTAAATGCTCCAAGTC
C37	TTTGTCGACCTTAAATTTCCTTGAGAAATTTTTTG
C38	GCACCGCTTTAACTTCTTCTAG
C44	TTGGATCCCATGCAAGAGCAGAATTTAAAG
C45	CACCCGGGTACCGAGAAAATAGTAATTTTTTCATTTTCTTATCC
C46	TTTTAGTACCTGGAGGGAATAATGGATATCACAATAGAATAAAAGGGC
C47	TTTGTCGACTTTTGTGCCATAGGCTTTG
C54	TTGAATTCTTTTGTGCCATAGGCTTTG
C98	GTTGCGAATTCATGGCTTCTTGTTGATCAGG
C99	CGTATTGCATGACATTGCACTCCACCGCTTCAGTTCATTTTGTCGGGAGTTC
C100	CGGAGAACGAGATGCAAAGATCTGATATAAGATATTATGAACTTG
C101	GGTCTGAATTCTAAAGCTTGCTTACCCTGTTC
C102	TAAAGATGGGGTTGCAGAAC
C103	AGATAATGCGCAAAACAATC
C113	GTACAGCCATGGGAATTCAAAGAGCATAAAGGCTTAAAATGATAAGATTAATATTAATATTAATTAACATTCTTTTTG
C114	ATGATCCTCGAGGAATTCTTACTCATAATGCTTAAATCCTAGATCTTTAAG
C115	GTACAGCCATGGGAATTCAAAGAGCATAAAGGCTTAAAATGTCTCTATGGAATTCTTTTTCTTACTC
C116	ATGATCCTCGAGGAATTCCTAAATCACAATCATAATGAAGGG
C117	GTACAGCCATGGGAATTCAAAGAGCATAAAGGCTTAAAATGAACAAAGCTTTTACTCTGCTTG
C118	ATGATCCTCGAGGAATTCTTATATCAGATCTTTGCATCTAGAACTTCC
C124	ATTGACGCTTTTGGAGAATG
C125	TAGGCATTTTACTCAATATCTTGCC
C126	GCAATTAGCGGTTATCACATAG
H11	GCAGCTCCATCAGCAAAAGGG
H12	TCCAACGTCATCTCGTTCTCCG
H13	GCTCGGTACCCGGGTGACTAAC
H14	TCCCCGGGTCATTATTCCCTC
H136	GAGCAGATCATCTCTGATCC
